# Supplementary material for: Self-supervised learning yields representational signatures of category-selective cortex
Source: Commun Biol. 2026 Aug 29;9:1153. doi: 10.1038/s42003-026-10843-3 (PMC13526027; doi:10.1038/s42003-026-10843-3)
Supplement: Supplementary file 1 [file 42003_2026_10843_MOESM1_ESM.pdf]

nature portfolio

Peer Review File

Self-supervised learning yields representational signatures of category-selective cortex

Corresponding Author: Dr Daniel Janini

This manuscript has been previously submitted at another journal. This document only contains information relating to versions considered at Communications Biology.

This file contains all reviewer reports in order by version, followed by all author rebuttals in order by version.

Version 0:

Reviewer comments:

Reviewer #1

(Remarks to the Author)
Summary
This paper includes an excellent synthesis of image datasets that comprehensively cover the representational structure that encompass the ventral visual stream. The test battery put forth by the authors will be of great use to the community, as many different hypotheses of high-level visual organization are brought together in one place. The authors test face and scene selectivity in multiple self-supervised ResNet-50 models trained on ImageNet and compare to a functional localizer fMRI experiment. Selectivity is rigorously defined as not only preference for a category, but generalization of that selectivity to images from other datasets. They provide evidence for there being very similar representational principles of face- and scene-selectivity in both models and the brain. They present this in support of domain-general learning mechanisms, as the similarity of these generic self-supervised models to the brain is existence proof that such category selectivity does not require highly specialized processes to emerge in a neural network representation. This is a rigorous, informative paper that really puts model-brain comparisons to the test and highlights the strength of these artificial neural networks for modelling high-level visual cortex. The below comments and suggestions for improvement should be addressed, to further strengthen the paper prior to publication.

Comments
Figure 1B, supplementary figures and lines 93 – 109:
The methods in Section 5.3 are well-described, but how the category-selective model units map to the single points plot in Figure 1B is unclear to me. There is a singular point per model/image set pair– is this the average activation across those model units? If so, then a measure of spread should be plot as well. If the error bars are so small that they are not visible here, then please include stats in the figure caption. Lines 105/106 describe the percentage of model selective units, but is there a way to interpret how these selective units relate? Are the two sets of units mutually exclusive, or is there overlap? Figure 1b convinces me of selectivity in model units for faces and for scenes, and the evidence for generalization across image sets is clear. However, Supplementary Fig. 1 is inconsistent with the in-text claim that “candidate category-selective units in earlier ReLU layers did not fully generalize across the test sets”. While true that within category generalization was not selective, I interpret this as candidate model units generalizing to other test set categories, just not strictly the within-category sets. This suggests that there is some other feature, that is likely common across these other categories and important to the earlier layers, that is driving the face selectivity. Is it possible that the z-scoring of the y axis is hiding key differences across the layers? This makes it appear as though earlier stages do have selectivity, but driven by another feature (for example, one that is common to Faces-Set1, Objects, Bodies-Set1 and Numbers in Face-selective units of Stage 1). This makes me wonder if the standardization is actually hiding the strength of your selectivity result, and some description of these potential differences would help with interpretation. Finally, it would be nice to make the range of the y axis consistent across Supplementary Fig. 1 (Stage 4) and Figure 1B.

Section 3.4.6 and Figure 5A inconsistencies:
The comparison between pareidolia images and their matched object counterparts is not depicted. Was a contrast done? The betas are plot in Figure 5A, but how this relates to the in-text result is unclear as this figure is not referenced in text. The figure caption reads “Right, responses in face-selective model units. Networks responded more to animal faces and

pareidolia faces than objects but not cartoon faces or eyes." However, the plot does not look consistent with this. It appears that the model face-selective units respond more to animal faces than the other categories, with perhaps a difference in eyes for some of the networks. Please amend and clarify upon review.

Section 3.4.5 and Figure 4:
Some discussion of how these results interact with the models' expected input distribution, given the training data, should be included. For these ImageNet trained models, the differences in activations between Empty Room and Mult. Furniture might be larger than expected than for the brain. Although this is what the results show, indeed there is some divergence between model and brain, I think further acknowledgement of these differences in terms of image statistics and out of distribution sampling is warranted. Furthermore, the Big Scene results are much lower in the networks – why is this? Is it something to do with the training distribution of ImageNet? Do models never see scenes like this? This should be acknowledged and discussed in text, especially in the context of Discussion lines 454-457.

Minor comments
Line 33: "Why do the FFA and PPA exhibit these distinct representational signatures?" sets it up as though you're testing if functional specificity exists for a certain purpose. Rather than asking why these signatures are present (in terms of a functional domain-specific goal, as you recognize is important in the discussion lines 477-486) your paper asks something more akin to: what drives the emergence of these distinct representational signatures in FFA and PPA?
Line 105/106 – The authors justifiably choose to focus on face and scene selective regions, but I do wonder were there any object selective units?
Figure 2C: The dashed line is a little confusing as I expected it to be the fMRI noise ceiling. The authors should consider including the noise ceiling across participants, as was reported in lines 152/3, and include the average correlation only as the sub-title.
Line 116/117 – "Thus, self-supervised training, rather than just architectural constraints, causes the emergence of robust category-selective features." Do you predict that supervised training, which you describe as domain-specific, would lead to the same result?
Reference 106: Appears to be incomplete.
Line 550: "in variance action poses" should likely be "in various action poses".

Reviewer #2

(Remarks to the Author)
This work addresses the important question whether category-selective regions (PPA and FFA) in visual cortex can emerge from domain-general learning mechanisms, rather than from mechanisms that are tailored to domain-specific task demands. To that end, the authors take self-supervised DNNs as a model of domain-general learning and quantify how well they are aligned with responses measured in human fMRI. First, the work applies a functional localizer approach to DNNs to identify face- and scene-selective populations. The authors then develop a very broad test battery of stimuli that have been used previously to characterize the function of PPA and FFA in neuroimaging studies. Responses to these images were measured in a group of human participants as well as the models, showing a high alignment between feature tuning. The manuscript goes into detail on the similarities and differences regarding commonly studied image features, including curvature, animacy, size, mid-level features, visual size tolerance, face-specific effects (e.g. pareidolia) and scene-specific effects (e.g. global layout vs. object content). In the vast majority of cases, the findings show a remarkable similarity between models and the human brain regarding feature-tuning in category-selective units/regions. In the cases where models are badly aligned, the manuscript clearly acknowledges this and discusses potential reasons. As far as I can tell, the methodology is rigorous.

Overall, these findings provide very comprehensive and nuanced evidence for the main claim of the authors: That domain-general, self-supervised learning mechanisms can produce category-selective representations for faces and scenes. I think the results will be highly relevant to the field, their breadth is compelling, and the chosen approach promises future advances beyond this work.

I really only see one major issue, which is the lack of an explicit alternative model. The findings provide ample support in favor of domain-general, self-supervised learning as a driver of category-selective regions. However, the alternative view outlined in the introduction is not really tested. As a result, the findings only support that domain-general learning is a sufficient explanation, but not whether it has more explanatory value than other accounts. The discussion section somewhat hints at this (line 477 and 509) but does not go into much detail. What if supervised networks (or whichever network might be chosen for comparison) contain face-/scene-selective units as well, with similar tuning characteristics? I see how a similarly comprehensive test might be difficult to achieve (especially within a revision). And of course the question which exact model would be best suited is difficult. However, this basic limitation could be acknowledged, if only as a cue for future research.

Sincerely,
Oliver Contier

Version 1:

Reviewer comments:

Reviewer #1

(Remarks to the Author)
I appreciate the authors' thorough responses and revisions. All of my comments have been addressed and I am satisfied with the current version of the manuscript.

Open Access This Peer Review File is licensed under a Creative Commons Attribution 4.0 International License, which permits use, sharing, adaptation, distribution and reproduction in any medium or format, as long as you give appropriate credit to the original author(s) and the source, provide a link to the Creative Commons license, and indicate if changes were made.
In cases where reviewers are anonymous, credit should be given to 'Anonymous Referee' and the source.
The images or other third party material in this Peer Review File are included in the article's Creative Commons license, unless indicated otherwise in a credit line to the material. If material is not included in the article's Creative Commons license and your intended use is not permitted by statutory regulation or exceeds the permitted use, you will need to obtain permission directly from the copyright holder.
To view a copy of this license, visit https://creativecommons.org/licenses/by/4.0/

We thank the reviewers for their time and constructive feedback. We hope that the following revisions adequately address the comments from both reviewers. Line numbers refer to the revised manuscript viewed in simple markup mode.

Reviewer #1 (Remarks to the Author):

Summary

This paper includes an excellent synthesis of image datasets that comprehensively cover the representational structure that encompass the ventral visual stream. The test battery put forth by the authors will be of great use to the community, as many different hypotheses of high-level visual organization are brought together in one place. The authors test face and scene selectivity in multiple self-supervised ResNet-50 models trained on ImageNet and compare to a functional localizer fMRI experiment. Selectivity is rigorously defined as not only preference for a category, but generalization of that selectivity to images from other datasets. They provide evidence for there being very similar representational principles of face- and scene-selectivity in both models and the brain. They present this in support of domain-general learning mechanisms, as the similarity of these generic self-supervised models to the brain is existence proof that such category selectivity does not require highly specialized processes to emerge in a neural network representation. This is a rigorous, informative paper that really puts model-brain comparisons to the test and highlights the strength of these artificial neural networks for modelling high-level visual cortex. The below comments and suggestions for improvement should be addressed, to further strengthen the paper prior to publication.

Thank you for your comments! We are glad you found the paper rigorous and informative!

Comments

Figure 1B, supplementary figures and lines 93 – 109:

The methods in Section 5.3 are well-described, but how the category-selective model units map to the single points plot in Figure 1B is unclear to me. There is a singular point per model/image set pair– is this the average activation across those model units?

In the caption for Fig 1B, we have clarified that each point represents the mean activation across model units: *"Each point represents the mean activation across the model units."*

If so, then a measure of spread should be plot as well. If the error bars are so small that they are not visible here, then please include stats in the figure caption.

Following your suggestion, we checked how the points would look with error bars. The error bars are very small and not clearly visible unless we increase the line width to a degree which introduces clutter between the points. Because of this, we have followed your suggestion of including stats in the caption instead.

Lines 105/106 describe the percentage of model selective units, but is there a way to interpret how these selective units relate? Are the two sets of units mutually exclusive, or is there overlap?

We have now clarified this point in lines 95-97: *"Because the selection criteria include a direct contrast between faces and scenes, the resulting sets of face- and scene-selective units are mutually exclusive."*

Figure 1b convinces me of selectivity in model units for faces and for scenes, and the evidence for generalization across image sets is clear. However, Supplementary Fig. 1 is inconsistent with the in-text claim that "candidate category-selective units in earlier ReLU layers did not fully generalize across the test sets". While true that within category generalization was not selective, I interpret this as candidate model units generalizing to other test set categories, just not strictly the within-category sets. This suggests that there is some other feature, that is likely common across these other categories and important to the earlier layers, that is driving the face selectivity. Is it possible that the z-scoring of the y axis is hiding key differences across the layers? This makes it appear as though earlier stages do have selectivity, but driven by another feature (for example, one that is common to Faces-Set1, Objects, Bodies-Set1 and Numbers in Face-selective units of Stage 1). This makes me wonder if the standardization is actually hiding the strength of your selectivity result, and some description of these potential differences would help with interpretation. Finally, it would be nice to make the range of the y axis consistent across Supplementary Fig. 1 (Stage 4) and Figure 1B.

Thank you for these points. Previously we summarized results in early model layers in one sentence. To improve clarity and consistency of our claims, we have now expanded this sentence to a short paragraph in lines 113-120. Previously when we wrote of "generalizing across the test sets", the type of generalization we had in mind was a maintenance of category-selectivity for the target category. We have changed the wording of this section to clarify this point. The paragraph can be read here:

> *"Consistent with previous research arguing for weaker category preferences in earlier model layers[49], we found that candidate category-selective units in earlier ReLU layers did not maintain selectivity for their target category across the test sets (Supplementary Fig. 1). Specifically, candidate face-selective units did not selectively exhibit higher responses for faces over all other categories. Likewise, candidate scene-selective units did not selectively exhibit higher responses for scene categories. Thus, in early model layers, units selected for their category preferences in one image set did not maintain selectivity for that category in the test sets."*

Your comments regarding z-scoring have made us aware that we did not adequately describe this aspect of our methods. We have added content to the methods section in lines 681-684 to explain and justify our z-scoring procedure:

> *"Neural network activations are expressed in arbitrary units and can differ in scale across units, so raw responses are not directly comparable. We therefore z-scored each unit's responses to place them on a common scale, ensuring equal contribution to mean response estimates."*

Lastly, we have incorporated your suggestion to make the y-axis consistent between Supplementary Figure 1 and Figure 1B.

Section 3.4.6 and Figure 5A inconsistencies:
The comparison between pareidolia images and their matched object counterparts is not depicted. Was a contrast done? The betas are plot in Figure 5A, but how this relates to the in-text result is unclear as this figure is not referenced in text. The figure caption reads "Right, responses in face-selective model units. Networks responded more to animal faces and pareidolia faces than objects but not cartoon faces or eyes." However, the plot does not look consistent with this. It appears that the model face-selective units respond more to animal faces than the other categories, with perhaps a difference in eyes for some of the networks. Please amend and clarify upon review.

We see how the writing in our previous draft was unclear on a few interrelated points.

First, we have now clarified that the object condition in this set of results was a set of objects matched to the pareidolia images. We have reworded lines 409-424 to clarify this point:

> *"Next, we tested signatures in the FFA related to face shape tuning. Face-selective cortex prefers faces across great texture variation, for example maintaining preferential responses for animal faces[15,16,65], illusory faces in objects (i.e., pareidolia)[17,78], and cartoon faces[15]. In addition, eyes alone are sufficient to drive preferential responses in the FFA, indicating a particular role for salient face parts[15]. To test these signatures in the FFA, we determined if responses to animal faces, pareidolia faces, cartoon faces, and eyes were all greater than responses to objects. For these comparisons, the object condition included images which had the same categories as the pareidolia faces, but which did not create an illusory percept of a face. All previous findings replicated (Fig 5A). In comparison to the object condition, the FFA exhibited a greater response to animal faces ($p < 10^{-4}$, two-tailed binomial test), pareidolia faces ($p < 10^{-4}$, two-tailed binomial test), cartoon faces ($p < 10^{-4}$, two-tailed binomial test), and eyes ($p < 10^{-4}$, two-tailed binomial test)."*

Second, thank you for pointing out that we forgot to include an in-text reference to Figure 5A. We have added it to Line 417 to clarify that we did conduct a contrast of pareidolia images and their matched objects. These changes ensure that the in-text description now matches the plotted contrasts in Fig. 5A.

Third, the previous wording in the caption for Figure 5 was confusing regarding which contrasts we were completing. We have changed the wording now to clarify that each face condition was contrasted with the object condition:

"Figure 5. A. Face specific conditions measuring sensitivity to face shapes across texture variation. Responses were measured to animal faces, pareidolia faces, objects matched to the pareidolia faces, cartoon faces, and eyes. Left, responses in the human FFA. The FFA responded more to all face conditions than pareidolia-matched objects. Right, responses in face-selective model units. Networks responded more to animal faces and pareidolia faces than pareidolia-matched objects. However, networks did not exhibit consistent preferences for cartoon faces or eyes in comparison to the object condition."

Section 3.4.5 and Figure 4:
Some discussion of how these results interact with the models' expected input distribution, given the training data, should be included. For these ImageNet trained models, the differences in activations between Empty Room and Mult. Furniture might be larger than expected than for the brain. Although this is what the results show, indeed there is some divergence between model and brain, I think further acknowledgement of these differences in terms of image statistics and out of distribution sampling is warranted. Furthermore, the Big Scene results are much lower in the networks – why is this? Is it something to do with the training distribution of ImageNet? Do models never see scenes like this? This should be acknowledged and discussed in text, especially in the context of Discussion lines 454-457.

We agree that this section could be improved by considering the image diet of the models. Thus, we have added content to this section of the results in response to your suggestions. First, we added the result that model units responded to big scenes less than other scene sizes (lines 396-400):

"Because we did not replicate previous fMRI findings that the PPA prefers big scenes over small scenes, we did not have strong predictions of what would constitute brain alignment in the scene-selective model units concerning variance among navigable scales of space. However, we note that scene-selective units responded to big scenes less than medium and small scenes ($p < 10^{-4}$, population prevalence testing)."

Second, we conducted an analysis of scene types in ImageNet. We find that empty rooms are relatively rare, and big scenes are underrepresented relative to the other scene sizes. We included these results in lines 401-407:

"Next, we conducted an exploratory analysis to test whether responses in scene-selective model units reflected the distribution of scene types present in the ImageNet training diet. In a random sample of 1000 images from ImageNet, 34.6% of the images depicted a navigable scene. Only 0.2% of the full sample depicted an empty room. Thus, partial preferences for empty room images emerged despite a relative paucity of these images in the training set. Big scenes were underrepresented (7.7%) relative to medium scenes (10.2%) and small scenes (16.7%)."

Lastly, in the discussion section we compare the image statistics of ImageNet to the image diet of human infants and suggest future studies that investigate the impact of image diet on scene representations (lines 486-491):

> *"our models exhibited a notable limitation: an incomplete preference for empty rooms and a bias instead toward arrays of objects. One possible explanation is the object-centric nature of the ImageNet training set, which rarely includes scene surfaces without object content. In contrast, the image diet of a human infant preferentially includes scene views with simple edge statistics such as walls, doors, and ceilings[98]. Future studies could test whether variations in image diet increase preferences for spatial layout information relative to object content."*

Minor comments

Line 33: "Why do the FFA and PPA exhibit these distinct representational signatures?" sets it up as though you're testing if functional specificity exists for a certain purpose. Rather than asking why these signatures are present (in terms of a functional domain-specific goal, as you recognize is important in the discussion lines 477-486) your paper asks something more akin to: what drives the emergence of these distinct representational signatures in FFA and PPA?

We agree that your suggested phrasing is closer to the central question we are asking, so we have edited lines 33-34 in accordance with this feedback: *"What drives the emergence of these distinct representational signatures?"*

Line 105/106 – The authors justifiably choose to focus on face and scene selective regions, but I do wonder were there any object selective units?

This is an interesting question. We do not think our dataset is well-equipped to answer questions about object-selective cortex, because it is usually identified in the brain using different stimulus conditions (for example, objects vs scrambled objects). However, we added the following to lines 528-530 in the discussion to alert readers to related work focusing on other categories:

> *"While we focused on faces and scenes due to their well characterized representational signatures, an emerging body of work leverages the localizer approach to investigate model representations of objects, written symbols, bodies, and tools[109–112]."*

Figure 2C: The dashed line is a little confusing as I expected it to be the fMRI noise ceiling. The authors should consider including the noise ceiling across participants, as was reported in lines 152/3, and include the average correlation only as the sub-title.

We have removed the dashed line in our plots so that it is not confused with the noise ceiling. We have added a line to the Figure 2C caption to indicate that the noise ceiling is the gray bar at the top of each plot.

Line 116/117 – "Thus, self-supervised training, rather than just architectural constraints, causes the emergence of robust category-selective features." Do you predict that supervised training, which you describe as domain-specific, would lead to the same result?

Yes, past research has shown that face- and scene-selective units also emerge in category-supervised networks. We now refer to this work in lines 80-82:

> *"In fMRI research, face- and scene-selective neural populations are typically identified using a localizer procedure that contrasts voxel responses to faces, scenes, and objects[2,4,52]. Recently, analogous procedures have revealed category-selective representations in category-supervised[42,53–55] and self-supervised neural networks[49–51]."*

Reference 106: Appears to be incomplete.

You are correct, and we have now fixed this reference.

Line 550: "in variance action poses" should likely be "in various action poses".

Thank you for reading our paper carefully. We have fixed this typo.

Reviewer #2 (Remarks to the Author):

This work addresses the important question whether category-selective regions (PPA and FFA) in visual cortex can emerge from domain-general learning mechanisms, rather than from mechanisms that are tailored to domain-specific task demands. To that end, the authors take self-supervised DNNs as a model of domain-general learning and quantify how well they are aligned with responses measured in human fMRI. First, the work applies a functional localizer approach to DNNs to identify face- and scene-selective populations. The authors then develop a very broad test battery of stimuli that have been used previously to characterize the function of PPA and FFA in neuroimaging studies. Responses to these images were measured in a group of human participants as well as the models, showing a high alignment between feature tuning. The manuscript goes into detail on the similarities and differences regarding commonly studied image features, including curvature, animacy, size, mid-level features, visual size tolerance, face-specific effects (e.g. pareidolia) and scene-specific effects (e.g. global layout vs. object content). In the vast majority of cases, the findings show a remarkable similarity between models and the human brain regarding feature-tuning in category-selective units/regions. In the cases where models are badly aligned, the manuscript clearly acknowledges this and discusses potential reasons. As far as I can tell, the methodology is rigorous.

Overall, these findings provide very comprehensive and nuanced evidence for the main claim of the authors: That domain-general, self-supervised learning mechanisms can produce category-selective representations for faces and scenes. I think the results will be highly relevant to the

field, their breadth is compelling, and the chosen approach promises future advances beyond this work.

Thank you for your positive feedback! We are happy to hear that you find the results comprehensive and compelling!

I really only see one major issue, which is the lack of an explicit alternative model. The findings provide ample support in favor of domain-general, self-supervised learning as a driver of category-selective regions. However, the alternative view outlined in the introduction is not really tested. As a result, the findings only support that domain-general learning is a sufficient explanation, but not whether it has more explanatory value than other accounts. The discussion section somewhat hints at this (line 477 and 509) but does not go into much detail. What if supervised networks (or whichever network might be chosen for comparison) contain face-/scene-selective units as well, with similar tuning characteristics? I see how a similarly comprehensive test might be difficult to achieve (especially within a revision). And of course the question which exact model would be best suited is difficult. However, this basic limitation could be acknowledged, if only as a cue for future research.

Sincerely,
Oliver Contier

We agree that our paper is limited to an exploration of self-supervised models, so we cannot make claims about whether this form of learning uniquely explains category-selective regions in comparison to other models. As you note, it remains possible that domain-specialized models could also yield face- and scene-selective units aligned with the brain. We have added a paragraph to the discussion section to more clearly address this limitation (see below).

In this paragraph, we outline how future studies could explore the space of domain-specialized learning pressures. As you point out, there are questions about what kinds of models are best suited for exploring domain-specialization, and comprehensively exploring this space is difficult. Therefore, in this limitation paragraph, we spell out the major factors that should be considered when operationalizing domain-specialized models (i.e., image diet, task, and finetuning operations) and relate these factors to the context of face and scene processing. A comprehensive comparison across this model space would require substantial additional work beyond the scope of the present study. However, we hope we have provided sufficient guidance on what investigating this model space would entail and drawn attention to the space of alternative models. This limitation paragraph (lines 538-551) can be read here:

“A limitation of this study is that we did not evaluate how domain-specific learning can shape category-selective representations. As such, our findings demonstrate that domain-general forms of learning are sufficient to produce many signatures of category-selective cortex, but they do not necessitate that this type of learning is uniquely capable of doing so. Competing neural network models could operationalize domain-specialized learning pressures through different factors[121]. One factor is image diet, for example training models exclusively on a specific domain (e.g., faces only) or with enriched exposure to certain categories (e.g., broader sampling of scene categories). Another factor is the learning objective itself, with multiple tasks to consider for each domain. For example, scene-related tasks could include categorization, navigational affordance prediction, or construction of surface geometries. Future studies could also compare purely domain-specialized models with models that combine self-supervised pre-training and domain-specialized finetuning. The test battery introduced in this paper provides shared evaluation criteria for comparing such models, paving the way to broader model comparison.”
