# Supplementary material for: Self-supervised learning yields representational signatures of category-selective cortex
Source: Commun Biol. 2026 Aug 29;9:1153. doi: 10.1038/s42003-026-10843-3 (PMC13526027; doi:10.1038/s42003-026-10843-3)
Supplement: Supplementary file 3 [file 42003_2026_10843_MOESM3_ESM.pdf]

nature portfolio

Corresponding author(s): Daniel Janini

Last updated by author(s): May 15, 2026

Reporting Summary

Nature Portfolio wishes to improve the reproducibility of the work that we publish. This form provides structure for consistency and transparency in reporting. For further information on Nature Portfolio policies, see our Editorial Policies and the Editorial Policy Checklist.

Statistics

For all statistical analyses, confirm that the following items are present in the figure legend, table legend, main text, or Methods section.

n/a	Confirmed	
☐	☒	The exact sample size (*n*) for each experimental group/condition, given as a discrete number and unit of measurement
☐	☒	A statement on whether measurements were taken from distinct samples or whether the same sample was measured repeatedly
☐	☒	The statistical test(s) used AND whether they are one- or two-sided<br>*Only common tests should be described solely by name; describe more complex techniques in the Methods section.*
☐	☒	A description of all covariates tested
☒	☐	A description of any assumptions or corrections, such as tests of normality and adjustment for multiple comparisons
☐	☒	A full description of the statistical parameters including central tendency (e.g. means) or other basic estimates (e.g. regression coefficient) AND variation (e.g. standard deviation) or associated estimates of uncertainty (e.g. confidence intervals)
☐	☒	For null hypothesis testing, the test statistic (e.g. *F*, *t*, *r*) with confidence intervals, effect sizes, degrees of freedom and *P* value noted<br>*Give P values as exact values whenever suitable.*
☒	☐	For Bayesian analysis, information on the choice of priors and Markov chain Monte Carlo settings
☒	☐	For hierarchical and complex designs, identification of the appropriate level for tests and full reporting of outcomes
☐	☒	Estimates of effect sizes (e.g. Cohen's *d*, Pearson's *r*), indicating how they were calculated

Our web collection on statistics for biologists contains articles on many of the points above.

Software and code

Policy information about availability of computer code

Data collection	The stimulus sets for data collection are available at https://osf.io/d9f5e/overview.
Data analysis	The code for all analyses is available at https://osf.io/d9f5e/overview.

For manuscripts utilizing custom algorithms or software that are central to the research but not yet described in published literature, software must be made available to editors and reviewers. We strongly encourage code deposition in a community repository (e.g. GitHub). See the Nature Portfolio guidelines for submitting code & software for further information.

Data

Policy information about availability of data

All manuscripts must include a data availability statement. This statement should provide the following information, where applicable:

- Accession codes, unique identifiers, or web links for publicly available datasets
- A description of any restrictions on data availability
- For clinical datasets or third party data, please ensure that the statement adheres to our policy

The fMRI dataset collected for this paper is available at https://openneuro.org/datasets/ds007368. The stimulus sets and code for all analyses are available at https://osf.io/d9f5e/overview.

Research involving human participants, their data, or biological material

Policy information about studies with human participants or human data. See also policy information about sex, gender (identity/presentation), and sexual orientation and race, ethnicity and racism.

Reporting on sex and gender	Here, we studied basic processes involved in visual perception and have no reason to believe sex or gender play a role.
Reporting on race, ethnicity, or other socially relevant groupings	We did not group participants by race, ethnicity, or any other socially relevant grouping.
Population characteristics	Neurotypical adults between the ages of 18-45 participated in the study.
Recruitment	Participants were recruited from the academic community and Berlin community through listserv advertisements. Participants were included who did not have mental illness and did not have metal implants. Given our recruitment methods, our dataset was biased to include healthy, young participants of higher educational status.
Ethics oversight	The ethical committee of the Department of Education and Psychology at Freie Universität

Note that full information on the approval of the study protocol must also be provided in the manuscript.

Field-specific reporting

Please select the one below that is the best fit for your research. If you are not sure, read the appropriate sections before making your selection.

☒ Life sciences ☐ Behavioural & social sciences ☐ Ecological, evolutionary & environmental sciences

For a reference copy of the document with all sections, see nature.com/documents/nr-reporting-summary-flat.pdf

Life sciences study design

All studies must disclose on these points even when the disclosure is negative.

Sample size	In this study, we replicate a wide number of previous findings from the literature. We included a sample of thirty-two participants, exceeding the sample sizes of previous studies which we replicated.
Data exclusions	Data were not excluded from the analysis
Replication	We have provided the fMRI data, code for reproducing analyses, and stimulus sets used when collecting the data. Thus, readers can replicate all findings from this paper. We have also reviewed the code to ensure replicability.
Randomization	Participants were not allocated into different experimental groups. All conditions were equally sampled in every participant.
Blinding	Blinding is not relevant here because participants were not allocated into different groups, and there is little that experimenters could do to bias the neural data in relation to the conditions.

Reporting for specific materials, systems and methods

We require information from authors about some types of materials, experimental systems and methods used in many studies. Here, indicate whether each material, system or method listed is relevant to your study. If you are not sure if a list item applies to your research, read the appropriate section before selecting a response.

Materials & experimental systems

n/a	Involved in the study	
☒	☐	Antibodies
☒	☐	Eukaryotic cell lines
☒	☐	Palaeontology and archaeology
☒	☐	Animals and other organisms
☒	☐	Clinical data
☒	☐	Dual use research of concern
☒	☐	Plants

Methods

n/a	Involved in the study	
☒	☐	ChIP-seq
☒	☐	Flow cytometry
☐	☒	MRI-based neuroimaging

Plants

Seed stocks | *Report on the source of all seed stocks or other plant material used. If applicable, state the seed stock centre and catalogue number. If plant specimens were collected from the field, describe the collection location, date and sampling procedures.*

Novel plant genotypes | *Describe the methods by which all novel plant genotypes were produced. This includes those generated by transgenic approaches, gene editing, chemical/radiation-based mutagenesis and hybridization. For transgenic lines, describe the transformation method, the number of independent lines analyzed and the generation upon which experiments were performed. For gene-edited lines, describe the editor used, the endogenous sequence targeted for editing, the targeting guide RNA sequence (if applicable) and how the editor was applied.*

Authentication | *Describe any authentication procedures for each seed stock used or novel genotype generated. Describe any experiments used to assess the effect of a mutation and, where applicable, how potential secondary effects (e.g. second site T-DNA insertions, mosiacism, off-target gene editing) were examined.*

Magnetic resonance imaging

Experimental design

Design type | The data set is task-based with a block design

Design specifications | Across the main experimental runs, each condition was measured five times in each participant.

We presented images in 16s blocks, with 20 images per block. We presented each image for 600ms with an overlaid fixation dot, and then we presented followed only the fixation dot for 200ms before the next image. We displayed images at 6.75 degrees of visual angle. Each run included four blocks with just the fixation dot, also lasting 16s each.

Behavioral performance measures | Button presses were recorded as participants performed a one-back task to ensure they were viewing the stimuli.

Acquisition

Imaging type(s) | Structural, Functional

Field strength | 3T

Sequence & imaging parameters | We collected data with a 64-channel head coil. We measured the anatomical image using a T1-weighted sequence (TR: 1.93s, TE: 3.52ms, number of slices: 256, voxel size: 0.8mm isotropic, FOV: 166.4mm x 237.03mm, flip angle: 8 degrees). We measured functional images with full brain coverage (TR: 2.0s, TE: 30.8ms, number of slices: 72, voxel size: 2.0mm isotropic, matrix size: 110 x 110, FOV: 220mm x 220mm, flip angle: 60 degrees, slice thickness: 2mm, inter-slice gap: 0mm, multiband factor: 3, acquisition order: interleaved).

Area of acquisition | The acquisition area included the whole brain

Diffusion MRI ☐ Used ☒ Not used

Preprocessing

Preprocessing software | We preprocessed fMRI data using fMRIPrep.

Normalization | Data were normalized to MNI space.

Normalization template | MNI152NLin2009cAsym

Noise and artifact removal | When estimating trial-level responses to each block, we conducted data-driven denoising with GLMSingle. We identified voxels whose activity did not relate to the experimental design, then we derived a set of noise regressors from their time courses using principal components analysis. We determined the ideal number of noise regressors to include by using cross-validation when fitting the general linear model.

Volume censoring | Volumes were not censored

Statistical modeling & inference

Model type and settings | We used GLMSingle to estimate response amplitudes for each block in each voxel in each participant. These trial-wise estimates were used to define functional regions of interest and to perform univariate analyses within these regions.

Effect(s) tested | Univariate analyses compared response magnitudes to groups of conditions within specific regions of interest.

Specify type of analysis: ☐ Whole brain ☒ ROI-based ☐ Both

Anatomical location(s)	Using data from the localizer runs, we identified the FFA and the PPA in each participant using a Group-Constrained Subject-Specific (GSS) procedure (see Julian et al., 2012)
Statistic type for inference (See Eklund et al. 2016)	Voxelwise and cluster-based methods were not used in this paper.
Correction	Not applicable, because voxelwise statistical tests were not conducted.

Models & analysis

n/a	Involved in the study
☒	☐ Functional and/or effective connectivity
☒	☐ Graph analysis
☒	☐ Multivariate modeling or predictive analysis
